# Phenolic Copigment Enhanced Anthocyanin Stability and Color Intensity of Frozen Red Huajiao (*Zanthoxylum bungeanum* Maxim.) Fruit

**DOI:** 10.3390/foods15101719

**Published:** 2026-05-13

**Authors:** Xin Yang, Yishan Chen, Xiao He, Jiani Liu, Shuang Xian, Shanshan Li, Xiaoyan Hou, Man Zhou, Qiang Cui, Jie Yu, Xiang Zhu, Zhiqing Zhang, Anjun Chen, Guanghui Shen

**Affiliations:** College of Food Science, Sichuan Agricultural University, Ya’an 625014, China; 13550232902@163.com (X.Y.); 15334585653@163.com (Y.C.); 18175033825@163.com (X.H.); 15242523815@163.com (J.L.); xianshuang@stu.sicau.edu.cn (S.X.); lss@sicau.edu.cn (S.L.); houxiaoyan106@163.com (X.H.); zhouman@sicau.edu.cn (M.Z.); cuiqiangwx@163.com (Q.C.); jieyu0609@163.com (J.Y.); zxpaocai@163.com (X.Z.); zqzhang721@163.com (Z.Z.); chen_anjun@sicau.edu.cn (A.C.)

**Keywords:** *Zanthoxylum bungeanum* Maxim., anthocyanins, phenolic acids, copigmentation, molecular docking, color stability

## Abstract

Anthocyanin stability substantially determines the postharvest storage quality of red Huajiao (*Zanthoxylum bungeanum* Maxim.). Herein, the composition of red Huajiao anthocyanins (RHAs) was characterized, and the copigmentation performance of seven phenolic compounds with RHAs was comparatively evaluated, together with verifying their practical efficacy in maintaining the overall quality of red Huajiao during frozen storage. UPLC-Q-TOF-MS/MS analysis identified ten anthocyanin monomers in RHAs, among which delphinidin-3,5-diglucoside (D3,5G, 28.23%), and delphinidin-3-O-glucoside (D3G, 14.86%) were verified as the predominant monomers. Naringin (NA) exhibited an optimal copigmentation effect, achieving a maximum color enhancement rate of 19.46% at a 1:40 molar ratio and a pH of 3.0 at 20 °C, while thermodynamic tests verified the excellent stability of the naringin–RHA complex. The copigmentation interactions between RHAs and copigments were largely attributed to hydrogen bonds, π–π stacking, and alkyl hydrophobic interactions. Considering practical application cost and flavor compatibility, chlorogenic acid (CGA) was selected as the preferred alternative copigment. Frozen storage tests suggested that soaking pretreatment with 10 mmol/L CGA effectively delayed color fading and maintained the integrity of the oil gland and the good sensory quality and color attributes of red Hujiao, with no adverse impacts on its inherent flavor and numbing components. Collectively, phenolic-mediated intermolecular copigmentation represents an efficient technical means for stabilizing color and maintaining the commercial quality of postharvest red Huajiao during frozen storage.

## 1. Introduction

*Zanthoxylum bungeanum* Maxim., a member of the Rutaceae family, is an economically important spice crop widely distributed across Asian countries, including China, India, Nepal, and Vietnam [1,2]. The fruit of *Z*. *bungeanum*, commonly known as red Huajiao in China, represents an indispensable ingredient in Sichuan cuisine due to its distinctive numbing sensation and aroma. These typical sensory properties are primarily attributed to alkylamides (e.g., hydroxy-α-sanshool) and volatile compounds such as limonene and linalool [3,4].

The characteristic bright red color of fresh red Huajiao is primarily derived from anthocyanins, which also contribute to its antioxidant and bioactive properties [5,6,7]. However, anthocyanins are chemically unstable and readily degrade under environmental stresses such as temperature, light, oxygen, and pH variations [8,9]. This instability inevitably causes rapid discoloration during postharvest storage, which greatly reduces the commercial quality of fresh red Huajiao. Conventional preservation strategies, such as drying, can prolong shelf life but often result in substantial losses of key aroma compounds and sensory quality [10,11,12]. With increasing consumer demand for minimally processed foods, maintaining both the color and flavor in fresh red Huajiao has become a critical challenge. Therefore, developing effective strategies to stabilize anthocyanins during storage is of considerable practical importance.

Among anthocyanin stabilization techniques, copigmentation is widely recognized as an effective strategy for enhancing anthocyanin stability in food matrices. This process involves non-covalent interactions between anthocyanins and copigments, typically phenolic compounds with π–conjugated structures. Multiple weak forces, including π–π stacking, hydrogen bonds, and hydrophobic interactions, can stabilize the flavylium cation structure of anthocyanins, thereby improving color intensity and reducing degradation rates. Mechanistically, copigmentation protects anthocyanins from nucleophilic attack by water molecules and inhibits the formation of colorless hydrated derivatives and chalcones, effectively slowing down color fading in food systems [13]. Numerous studies have confirmed that phenolic acids and flavonoids can effectively enhance anthocyanin stability in different food models. For instance, Chen et al. [14] reported that chlorogenic acid and ferulic acid significantly improved the thermal stability and color retention of cyanidin-3-O-glucoside from mulberry anthocyanins. Sendri et al. [15] found that naringin exerted the strongest copigmentation effect on cyanidin-3-O-arabinoside in citrus peel juice. Similarly, Lin et al. [16] demonstrated that gallic acid supplementation effectively improved the color performance of purple cabbage anthocyanin beverages and prolonged the pigment degradation half-life to 14.87 days. These findings collectively suggest that the effectiveness of copigmentation is highly dependent on the structural characteristics of both anthocyanins and copigments. Although copigmentation mechanisms have been well explored in common fruits and vegetables, targeted investigations focusing on red Huajiao anthocyanins (RHAs) remain limited. Red Huajiao is a characteristic spice rich in delphinidin-based anthocyanins with special structural characteristics, and its copigmentation response to diverse phenolic copigments cannot be directly inferred from existing plant models. Furthermore, few studies have thoroughly integrated spectroscopic characterization, thermodynamic parameters, molecular docking simulation, and practical frozen storage experiments to elucidate the relationships among molecular structure, interaction mechanisms, and preservation efficacy.

Therefore, the objectives of this study were to (i) characterize the chemical composition of RHAs using UPLC-Q-TOF-MS/MS, (ii) systematically evaluate the copigmentation effects of seven common phenolic compounds, including naringin (NA), rosmarinic acid (RA), chlorogenic acid (CGA), caffeic acid (CFA), ferulic acid (FA), gallic acid (GA), and coumaric acid (CA), (iii) explore how pH and storage conditions affect the stability of copigmented RHA complexes, (iv) elucidate the underlying interaction mechanisms using FT-IR spectroscopy and molecular docking, and (v) verify the potential of selected copigments to delay color deterioration and improve the overall postharvest storage quality of fresh red Huajiao under frozen conditions. This study attempts to provides reliable theoretical support and practical technical references for enhancing color stability and extending the shelf life of anthocyanin-rich agricultural spices during frozen storage.

## 2. Materials and Methods

### 2.1. Materials

Fresh red Huajiao samples were harvested in August 2024 from Hanyuan County, Ya’an city, Sichuan Province, China. Seven phenolic copigments, including NA, RA, CGA, CFA, FA, GA, and CA were purchased from Shanghai Aladdin Biochemical Technology Co., Ltd. (Shanghai, China). Two standard anthocyanin monomers, delphinidin-3,5-diglucoside (D3,5G, ≥95% purity), delphinidin-3-O-glucoside (D3G, ≥95% purity), and citric acid–sodium citrate buffer (0.1 M, pH 3.0) were obtained from Shanghai Yuanye Bio-Technology Co., Ltd. (Shanghai, China). All other analytical grade chemical reagents were purchased from Chengdu Kelong Chemical Co., Ltd. (Chengdu, China).

### 2.2. Composition Analysis of RHAs

#### 2.2.1. Preparation of RHA Samples

The dried pericarp of red Huajiao was dried, ground, and sieved through a 60-mesh screen to obtain a uniformly fine powder. Briefly, 3.0 g of the prepared powder was accurately weighed and homogenously mixed with 90 mL of 60% (*v*/*v*) ethanol. The resulting mixture was subjected to ultrasonic-assisted extraction using a KH-300DE ultrasonic cleaner (Hechuang Ultrasonic Instrument Co., Ltd., Kunshan, Jiangsu, China) under controlled conditions: 25 °C, 40 kHz frequency, 270 W power, and a duration of 40 min. After extraction, the mixture was centrifuged at 10,000 r/min for 20 min at 4 °C using a ST16R centrifuge (Thermo Fisher Scientific, Waltham, MA, USA). The supernatant was collected and concentrated using rotary evaporation for 40 min to obtain crude RHA extract. For further purification, 20 mL of the crude extract was loaded onto a D-101 macroporous resin column (Φ1.6 cm × 70 cm). After 12 h of static adsorption, the column was eluted with 60% (*v*/*v*) ethanol at a constant flow rate of 1 mL/min. The eluted fractions were combined and concentrated using vacuum rotary evaporation at 40 °C for 40 min to remove residual ethanol. Finally, the purified solution was freeze-dried for 48 h using a Scientz-12N freeze dryer (Ningbo Scientz Biotechnology Co., Ltd., Ningbo, Zhejiang, China) to obtain RHA powder for subsequent experiments. The total anthocyanin content was quantified using the pH gradient method, and the anthocyanin purity was calculated using Equation (1).

(1) $$B/\%=\frac{\rho \times V}{m}\times 100$$

where *ρ* represents the detected anthocyanin concentration (g/L), *V* denotes the solution volume (L), and *m* indicates the mass of the tested RHA sample (g).

#### 2.2.2. Characterization of Anthocyanin Monomers

The anthocyanin monomers in purified RHAs were separated and identified using an Agilent 6530 Q-TOF LC-MS (Agilent Technologies Co., Ltd., Milford, AS, USA). Semi-quantitative analysis of anthocyanin monomers was conducted based on relative peak areas, in accordance with previously described analytical protocols by Chen et al. [14] and Huang et al. [17].

Chromatographic separation was performed on a UPLC CSH C18 column (2.1 mm × 100 mm, 1.7 μm, Waters, Milford, MA, USA) maintained at 30 °C. The mobile phase consisted of 0.1% formic acid aqueous solution (phase A) and pure methanol (phase B). The gradient elution procedure was programmed as follows: 0–30 min, 5–25% B; 30–35 min, maintained at 25% B; 35–40 min, 25–100% B; 40–45 min, returned to 5% B. The flow rate was kept constant at 0.3 mL/min throughout the entire run, with an injection volume of 2.0 μL. The instrument was equilibrated for 5 min before the injection of each subsequent sample to ensure system stability and reproducible baseline conditions.

Untargeted mass spectral acquisition was performed in full scan mode over the mass range of *m*/*z* 100–2000, with both electrospray ionization positive (ESI+) and negative (ESI−) modes applied. Nitrogen was employed as the curtain gas (CUR) at a pressure of 35 psi, while the ion source gases (GS1 and GS2) were both set to 50 psi. The ion source temperature was maintained at 500 °C, and the ion spray voltage was set to 5500 V in positive mode and −4500 V in negative mode. The MS/MS operational parameters were configured as follows: declustering potential of 80 (+)/−80 (−), collision energy of 45 (+)/−45 (−), and a collision energy spread of 15 V. Instrument mass accuracy was calibrated using APCI positive/negative calibration solutions (AB Sciex, Marlborough, MA, USA).

Anthocyanin monomers were structurally identified according to their molecular ion profiles and characteristic secondary fragment ions [18]. The obtained mass fragmentation patterns were further cross-referenced with published databases and spectral information from Wang et al. [19] and Wu and Prior [20], enabling the reliable assignment of aglycone structures and the corresponding molecular weights of target anthocyanins.

### 2.3. Effect of RHA–Copigment Molar Ratio on the Copigmentation Effects and Thermodynamic Parameters of RHAs

#### 2.3.1. Absorption Spectra of Copigmentation Solutions

The RHA–copigment solutions were prepared following the method reported by Sendri et al. [15]. Briefly, the purified RHA powder was fully dissolved in a citric acid–sodium citrate buffer (0.1 M, pH 3.0) to obtain an anthocyanin working solution at a concentration of 2.5 × 10^−4^ mol/L. Seven structurally diverse copigments, namely NA, RA, CGA, CFA, FA, GA, and CA, were separately dissolved in a phosphate buffer solution (0.06 M, pH 3.0) supplemented with 12.5% ethanol to generate a series of copigment stock solutions at gradient concentrations of 0.25, 1.25, 2.5, 5, and 10 mmol/L. Copigmentation reaction systems with molar ratios of RHA to copigment ranging from 1:1 to 1:40 (1:1, 1:5, 1:10, 1:20, and 1:40) were constructed by mixing equal volumes of the RHA working solutions and copigment stock solutions. All mixed samples were incubated in the dark at 20 °C for 30 min to reach thermodynamic equilibrium prior to spectral analysis. Preliminary pre-experiments verified that no further changes in absorbance occurred with extended incubation, confirming that the RHA–copigment complexes were fully and stably formed within 30 min. Subsequently, the prepared equilibrium reaction solutions were scanned using a 759S UV–Vis spectrophotometer (Shanghai Lengguang Technology Co., Ltd., Shanghai, China) over the wavelength range of 450–700 nm, and the corresponding absorption profiles were recorded. The absorbance of each sample solution was measured at 525 nm. The copigmentation effect was evaluated by calculating the copigmentation enhancement amplitude (ΔA) and the bathochromic shift of the maximum absorption wavelength (Δλ_max_), as recommended in a previous study [21]. The copigmentation effect rate (ΔA) was calculated using Equation (2), and the bathochromic shift value Δλ_max_ was determined using Equation (3).

(2) $$\Delta A(\%)\;=\frac{A-A_{0}}{A_{0}}\;\times \;100$$

where A_0_ is the absorbance of the pure RHA solution without copigments at 525 nm and A is the absorbance of the corresponding RHA–copigment complex solution at 525 nm.

Δλ_max_ = λ_maxc_ − λ_max0_ (3)

where λ_max0_ is the maximum absorption wavelength of the pure RHA solution and λmaxc is the maximum absorption wavelength of the RHA–copigment complex solution.

#### 2.3.2. Effect of Copigmentation on the Color Stability of RHAs

Seven copigment solutions and the RHA aqueous solution were freshly prepared according to the protocols described in Section 2.3.1, with fixed concentrations of 10.0 mmol/L and 0.25 mmol/L, respectively. Subsequently, 2.0 mL of individual copigment solution and 4.0 mL of the preprepared RHA working solution were accurately pipetted and fully blended in centrifuge tubes. All mixed systems were thoroughly vortexed and incubated at 25 °C for 30 min under dark conditions to reach stable equilibrium. After incubation, the absorption spectrum of each sample was immediately scanned over the wavelength range of 450–700 nm for subsequent spectral analysis.

#### 2.3.3. Determination of Thermodynamic Parameters

The key thermodynamic parameters of the copigmentation interaction, including the equilibrium constant (K) and stoichiometric ratio (n), were calculated based on the linear fitting results derived from Equation (4), and the Gibbs free energy (ΔG) was further quantified using Equation (5).

(4) $$\ln(\frac{A-A_{0}}{A_{0}})=\ln K+{n\;\times \;\ln\left(C_{p}\right)}_{0}$$

(5) ΔG=−RTlnK

where A_0_ refers to the absorbance of pure RHA solution without copigment at 525 nm, A is the absorbance value of RHA–copigment mixtures with gradient copigment concentrations at 525 nm after 30 min of dark incubation, n represents the binding stoichiometric ratio between copigment and anthocyanin, (Cp)_0_ denotes the initial molar concentration of the phenolic copigment, R is the universal gas constant (8.314 J·mol^−1^·K^−1^), T is the Kelvin temperature (K) of the reaction system, and *K* is the thermodynamic equilibrium constant of the copigmentation reaction.

### 2.4. Effect of Different pH Conditions on the Copigmentation of RHAs

For subsequent pH stability evaluation, RHA–copigment mixed solutions were uniformly prepared at a fixed molar ratio of 1:40 (RHA to copigment), calculated based on the extract-equivalent molar concentration. The pH of each prepared reaction system was accurately adjusted to a gradient range of 2.0–5.0 using 1 mol/L HCl or 1 mol/L NaOH. The resultant spectral variations were monitored by scanning the absorption spectra of all model samples across the wavelength range of 450–700 nm using a 759S UV–Vis spectrophotometer (Shanghai Lengguang Technology Co., Ltd., Shanghai, China).

### 2.5. Analysis of Color Stability of RHA–Copigment Complexes During Storage

Considering the complex chemical composition of natural RHAs, two dominant anthocyanin monomers with high relative abundance, D3G and D3,5G were selected as representative target components for the storage stability evaluation. All RHA–copigment solutions were freshly prepared at a fixed molar ratio of 1:40 (RHA to copigment) following the detailed protocol described in Section 2.3.1. The prepared samples were sealed and incubated in a constant-temperature incubator at 25 °C under dark conditions to simulate ambient storage environments. The color parameters, L*, a*, and b* values were periodically measured at 5-day intervals using a SC-10 portable colorimeter (Shenzhen ThreeNH Technology Co., Ltd., Shenzhen, Guangdong, China). The total color difference (Δ*E*) was calculated according to Equation (6) [22].

(6) $$\Delta E=\sqrt{{\left(L^{*}-L_{0}^{*}\right)}^{2}+{\left(a^{*}-a_{0}^{*}\right)}^{2}+{\left(b^{*}-b_{0}^{*}\right)}^{2}\;}$$

where Δ*E* denotes the total color difference, $L_{0}^{*}$, $a_{0}^{*}$, and $b_{0}^{*}$ correspond to the initial lightness, red–green chromaticity, and yellow–blue chromaticity values of RHA–copigment solution measured at day 0; L*, a*, and b* represent the real-time color parameters determined at each sampling time point during storage.

### 2.6. FT-IR Analysis

FT-IR spectroscopy was employed to characterize variations in functional groups and clarify the intermolecular interaction mechanisms between RHAs and selected phenolic copigments. The RHA–copigment mixture was prepared according to the method described in Section 2.3.1, with a molar ratio of 1:40 at pH 3.0. The well-mixed reaction systems were freeze-dried into solid powders and then thoroughly ground with potassium bromide (KBr) at a mass ratio of 1:200. After homogeneous grinding, the mixture was pressed into transparent pellets for spectroscopy records. The FT-IR spectra were collected using a Nicolet iS10 FT-IR spectrophotometer (Thermo Fisher Scientific, Waltham, MA, USA) over the wavenumber range of 4000 to 400 cm^−1^ at a resolution of 4 cm^−1^ and with a total of 32 scans.

### 2.7. Molecular Docking

The binding affinity and conformation of the copigmentation complexes between dominant anthocyanin monomers (D3G and D3,5G) and copigments were predicted using molecular docking techniques. The two anthocyanin monomers and seven copigment molecules were structurally retrieved from the PubChem database (https://pubchem.ncbi.nlm.nih.gov/). All molecular geometries were energy-minimized using Chem3D software (Version 23.1.1.3), followed by format conversion and export in PDB format for subsequent computation. The flexible molecular docking simulations between anthocyanin receptors and phenolic copigments were efficiently implemented via AutoDock Vina (version 1.2.0). After docking calculation, the optimal binding poses and intermolecular interaction profiles were imported into PyMol 3.1.0 for visual observation and structural characterization of RHA–copigment complex configurations. To better simulate the actual experimental conditions, the protonation states of all molecular structures were pre-adjusted to pH 3.0, consistent with the acidic copigmentation reaction environment in vitro. Implicit water solvent parameters were activated throughout the computational process to accurately simulate real aqueous solution microenvironments. Furthermore, a redocking strategy was conducted for protocol validation: the original ligand conformation was extracted and redocked into the defined binding region, and the RMSD value between the redocked pose and the initial conformation was calculated. A RMSD value below 2.0 Å verified the stability, accuracy, and reliability of the established docking protocol and parameter settings.

### 2.8. Effect of CGA Treatment on the Quality of Frozen Red Huajiao

#### 2.8.1. Sample Pretreatment with CGA Immersion

CGA was selected as the optimal natural copigment for fresh red Huajiao pretreatment, considering its excellent copigmentation performance, low cost, good economic applicability, and negligible adverse effects on the intrinsic flavor and taste characteristics of spices. Freshly harvested red Huajiao was immediately transported to the laboratory within 2 h. Fresh red Huajiao with intact pericarp, undamaged oil glands, and no signs of rot or mold was manually selected for subsequent experiments. All qualified fresh samples were pre-cooled at 4 °C to rapidly remove field heat and stabilize the initial physiological quality. CGA powder was fully dissolved in pre-cooled drinking water at 4 °C to prepare gradient working solutions with concentrations of 5.0, 10.0, and 15.0 mmol/L. The pre-cooled red Huajiao samples were immersed in each CGA solution for 2 min, gently taken out, and naturally air-dried at ambient temperature to remove residual surface moisture. Afterwards, the samples were evenly divided and sealed in aluminum foil bags (18 × 26 cm), with 100 g of red Huajiao per bag. Vacuum sealing was performed under a pressure of 0.07 MPa for 18 s to remove oxygen and avoid secondary contamination. All packaged samples were immediately transferred to a −18 °C constant-temperature freezer for long-term frozen storage. Corresponding experimental groups were labeled as CGA-5, CGA-10, and CGA-15 according to CGA treatment concentrations. Samples immersed in pure, pre-chilled drinking water (4 °C) for 2 min without CGA addition were set as the blank control group.

#### 2.8.2. Quality Analysis of Stored Red Huajiao

The red Huajiao samples were randomly collected from each treatment group at 7-day intervals during the 35-day frozen storage period for quality monitoring. In sum, one bag of samples was separated into individual grains at 4 °C to maintain fresh physical conditions before testing. The overall visual appearance of the samples was observed and photographed, and the surface color attributes and the total pungent substances content were measured.

##### Sensory Evaluation

The sensory evaluation of red Huajiao referred to He et al. [23] with minor modifications. Briefly, 10 g of the red Huajiao grains from each group were randomly sampled, coded with random numbers, and placed in Petri dishes at room temperature. A sensory evaluation panel consisting of 10 well-trained panelists aged 20–25 years was organized to conduct all sensory tests in a double-blind and randomized sequence to avoid subjective interference. Ethical approval and informed consent from all panel participants were obtained prior to the experiment. The sensory evaluation focused on the color, flavor, and oil gland integrity of the red Huajiao, and the specific scoring standards are listed in Table S1. The individual scores of the three sensory attributes and the overall acceptability were statistically averaged for subsequent comprehensive comparison.

##### Color Determination

The surface color characteristics of red Huajiao samples were analyzed using an IRIS electronic visual analyzer (Alpha M.O.S Co., Ltd., Toulouse, France). Prior to image acquisition, samples were uniformly placed in the center of a standard white background plate to ensure consistent shooting conditions. The instrumental pixel resolution was set to 2588 × 1942, and both top and bottom light sources were simultaneously turned on to achieve uniform and shadow-free illumination. The effective color feature data were automatically extracted from the central circular region of each collected image. Colorimetric parameters including L*, a and b values, and RGB optical information were obtained to comprehensively reflect the surface color variation of red Huajiao during frozen storage. A color change rate greater than 1% was adopted for subsequent statistical analysis, while data below this threshold were considered instrumental background noise and lacked statistical significance.

##### Flavor Analysis Using Electronic Nose (E-Nose)

The flavor profiles of red Huajiao samples were analyzed using the method reported by He et al. [23] with some modifications. A CNose electronic nose (Baosheng Industrial Development Co., Ltd., Shanghai, China) equipped with a multi-sensor array was employed to distinguish the comprehensive odor fingerprints of all treatment groups, with each sample tested in triplicate. Prior to measurement, residual gases were purged and clean air treated with activated carbon was introduced into the sensor chamber for 30 min to ensure stable sensor signals. Then, 2.0 g of red Huajiao sample was accurately weighed and transferred into a 15 mL headspace vial, which was immediately sealed and statically equilibrated at ambient temperature for 60 s prior to detection. The purified air served as the carrier gas at a constant flow rate of 400 mL/min, the sensor self-purging time was 120 s, and the data acquisition duration was 60 s. The sensor array contains 11 metal oxide sensors with specific response selectivity to different volatile categories. Specifically, sensors S6, S9, S15, and S18 are highly sensitive to aldehydes, ketones, and alcohols, sensors S4 and S16 respond to sulfur-containing compounds, sensors S5 and S17 are responsible for nitrogen-containing compounds, and sensors S1, S2, and S14 mainly captured hydrocarbon components. All raw sensor response data were further corrected using baseline calibration and unified normalization pretreatment to eliminate instrumental systematic errors and environmental background interference for subsequent multivariate statistical analysis.

##### Quantification of Total Pungent Substances

The content of total pungent substances in red Huajiao samples was determined according to the method of Tao et al. [24] with slight modifications. Briefly, 5.0 g of homogenized red Huajiao sample was accurately weighed and mixed with 50.0 mL of methanol, followed by 30 min ultrasound-assisted extraction at a frequency of 100 Hz and a power of 270 W. The obtained extract was filtered into a 100 mL brown volumetric flask, diluted to the constant volume, and mixed thoroughly. Thereafter, 100 μL of the above diluted solution was accurately pipetted into a 50 mL brown volumetric flask and diluted to the constant volume using ultrapure water. The absorbance of the final diluted solution was measured at 270 nm using a UV spectrophotometer. The total pungent substance content (mg/g) was quantitatively calculated based on the standard curve established with a 95% hydroxy-α-sanshool standard. The corresponding linear regression equation was Y = 53.965X − 0.004, with a high determination coefficient of *R*^2^ = 0.9995.

### 2.9. Statistical Analysis

All results are presented as the mean ± standard deviation of at least three independent replicates. Data analysis was conducted using SPSS Statistics 27.0 (IBM Corp., Armonk, NY, USA), and graphical visualization was performed with OriginPro 2022 (OriginLab Corp., Northampton, MA, USA). One-way analysis of variance (ANOVA) was applied, followed by the least significant difference (LSD) test for post-hoc comparisons. PLS-DA was carried out using SIMCA 14.1 (Umetrics, Umea, Sweden), and variable importance in the projection (VIP) scores were calculated. Differences were considered statistically significant at *p* < 0.05.

## 3. Results and Discussion

### 3.1. Chemical Composition of RHAs

**Figure 1:**
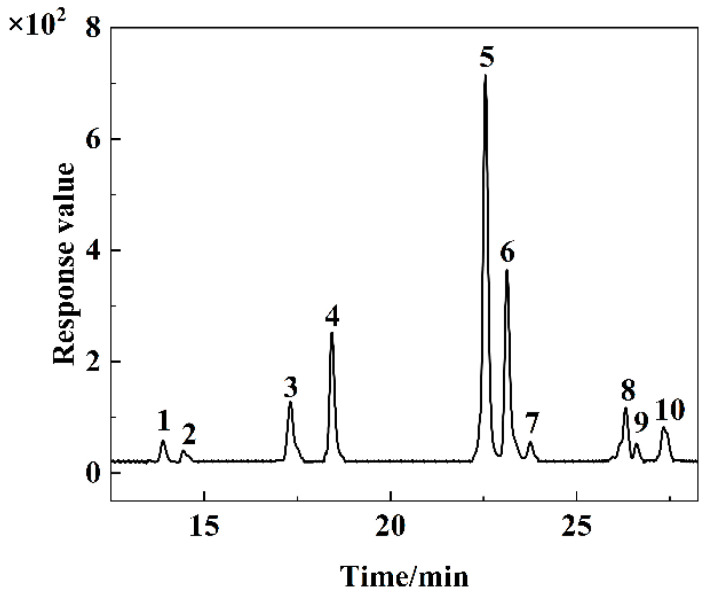
UPLC-Q-TOF-MS/MS plot of RHAs.

The total anthocyanin content of purified RHAs was determined as 100.19 ± 14.20 mg/g. The anthocyanin profile was further characterized using UPLC-Q-TOF-MS/MS. The total ion chromatogram is displayed in Figure 1, and detailed information including the retention time, molecular ions, fragment ions, and relative abundances of anthocyanin monomers is summarized in Table 1. A total of ten anthocyanin monomers were successfully identified, consisting of six delphinidin derivatives, two petunidin derivatives, and two cyanidin derivatives. The six delphinidin-type anthocyanins were annotated as delphinidin-3,5-diglucoside (*m*/*z* 627.15 → 465.11/303.05, D3,5G), delphinidin-3-O-glucoside (*m*/*z* 465.10 → 303.05, D3G), delphinidin-3-O-rutinoside-7-O-glucoside (*m*/*z* 773.21 → 611.16/465.10/303.05, D3R7G), delphinidin-3-O-rutinoside (*m*/*z* 611.16 → 465.10/303.05, D3OR), delphinidin-3-O-rhamnoside (*m*/*z* 449.11 → 303.05, D3R), and delphinidin-3-O-galactoside (*m*/*z* 465.10 → 303.05, D3OG). The petunidin derivatives included petunidin-3-O-rutinoside (*m*/*z* 625.18 → 479.12/317.07, P3R) and petunidin-3-O-glucoside (*m*/*z* 479.11 → 317.06, P3G), whereas the cyanidin derivatives were cyanidin-3-O-rutinoside (*m*/*z* 585.17 → 287.05, C3R) and cyanidin-3-O-glucoside (*m*/*z* 449.10 → 287.05, C3G). The corresponding MS/MS secondary fragmentation spectra and proposed structures are provided in Figure S1. Quantitative analysis showed that delphinidin derivatives dominated the anthocyanins in RHAs, accounting for 80.84% of the total relative content. Among these, D3,5G and D3G were the most abundant anthocyanin monomers, representing 28.23% and 14.86%, respectively. Notably, the anthocyanin fingerprint of the Hanyuan-sourced red Huajiao characterized in the present study differed distinctly from that of previous reports. For instance, Zheng et al. [25] reported peonidin-3-O-glucoside as the major anthocyanin monomer in red Huajiao from Yangling, Shaanxi province, while Wang et al. [26] reported cyanidin-based anthocyanins (cyanidin-3-O-galactoside, cyanidin-3-O-rhamnoside, and cyanidin-3-O-glucoside) as dominant monomers in the Dahongpao cultivar from Feng County, Shaanxi province. These compositional discrepancies may be potentially associated with differences in cultivar genotype, geographical cultivation regions, and climatic environmental conditions, all of which are well-established key factors modulating in vivo biosynthesis and the metabolite accumulation of anthocyanin. Structurally, delphinidin derivatives possess higher B-ring hydroxylation degrees compared with petunidin and cyanidin analogs. Therefore, it is hypothesized that their predominance in RHAs could potentially contribute to enhanced intermolecular interaction potential, which may in turn influence subsequent copigmentation behavior and color stability.

### 3.2. Copigmentation Effects of Different Copigments on RHAs

**Figure 2:**
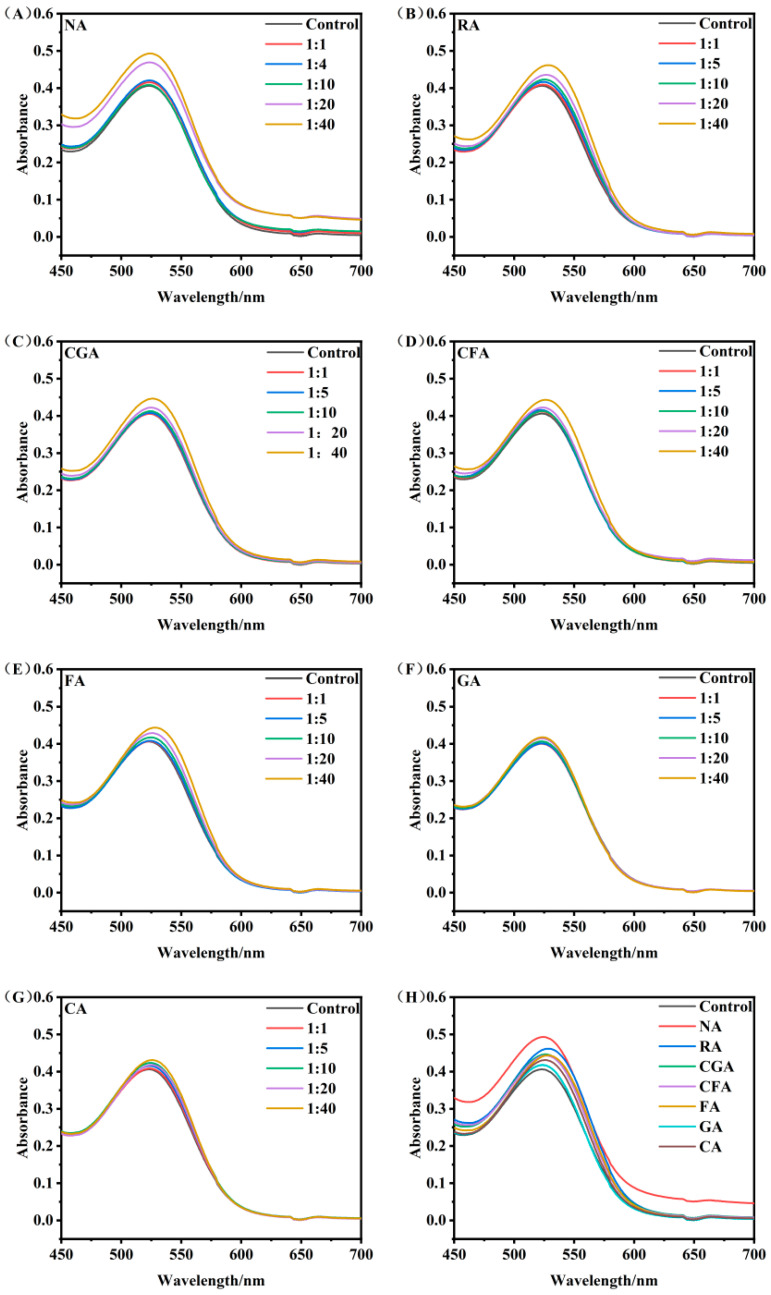
Visible absorption spectra of RHA–copigment complexes with seven copigment compounds at different molar ratios. (**A**) NA, (**B**) RA, (**C**) CGA, (**D**) CFA, (**E**) FA, (**F**) GA, (**G**) CA, and (**H**) the comparison copigmentation effect of seven copigments at 1:40 molar ratio.

As shown in Figure 2, supplementation with the seven selected phenolic copigments substantially modified the visible spectral characteristics of RHAs within the range of 450–700 nm. Compared with the control group without copigments, all treated samples exhibited increased absorbance intensity accompanied by an obvious bathochromic shift in the maximum absorption wavelength (λ_max_), indicating the formation of RHA–copigment copigmentation complexes. As summarized in Table 2, the copigmentation performance presented a positive concentration-dependent correlation with copigment dosage. At a molar ratio of 1:40 (RHA/copigment), the absorbance at 525 nm increased by 4.91–19.46%, and the corresponding bathochromic λmax shift ranged from 1 nm to 5 nm. These results suggest that the copigmentation reaction of RHAs was significantly enhanced with increasing copigment concentration, which is consistent with previously reported anthocyanin copigmentation patterns [27,28]. The enhanced color intensity and pectral redshift were mainly attributed to the increased formation of non-covalent complexes under high copigment dosages. Notably, under identical molar concentration conditions, distinct differences in copigmentation efficiency were observed among the seven phenolic compounds (Figure 2H). Specifically, at the molar ratio of 1:40, the copigmentation capacity followed the order: NA > RA, CGA > FA > CFA, GA, CA (*p* < 0.05). Such evident efficiency discrepancies indicated that the copigmentation capacity was inherently determined by the structural characteristics of phenolic copigments. Previous studies have suggested that anthocyanin copigmentation is largely influenced by the non-covalent forces and hydrophobic forces between aromatic skeletons and flavylium cations [29]. Copigment molecules with larger π-conjugated systems and multiple aromatic rings might provide stronger stacking forces, thereby effectively improving anthocyanin color stability and copigmentation efficiency. In this study, structural comparison showed that both NA and RA possessed dual aromatic ring structures, while CGA contained one aromatic ring combined with an additional six-membered heterocyclic structure, whereas the remaining four phenolic compounds contained only a single aromatic ring. This structural difference was highly consistent with the observed copigmentation efficiency ranking. Moreover, the number of unsaturated C=C double bonds in molecular skeletons also positively regulated the intermolecular binding strength [30]. In particular, the flavonoid C-ring of NA contained abundant conjugated double-bond structures, further strengthening its intermolecular affinity toward RHA molecules and thus achieving the optimal copigmentation effect. Overall, these results suggest that preliminary structure-related trends, in which the copigmentation efficiency of phenolic compounds appears to be governed by the enhanced combined effects of π-conjugation and functional group availability, may influence the strength and stability of non-covalent interactions with anthocyanins. It is worth noting that the above structural speculation and mechanism analysis were primarily derived from spectroscopic data obtained in simplified model solution systems. Such controlled laboratory conditions exclude the complex endogenous components in real red Huajiao matrices, such as polysaccharides, lipids, and proteins, which may interfere with anthocyanin–copigment interactions.

### 3.3. Enhanced Copigmentation Effect of NA Combined with Six Phenolic Acids on RHAs

**Figure 3:**
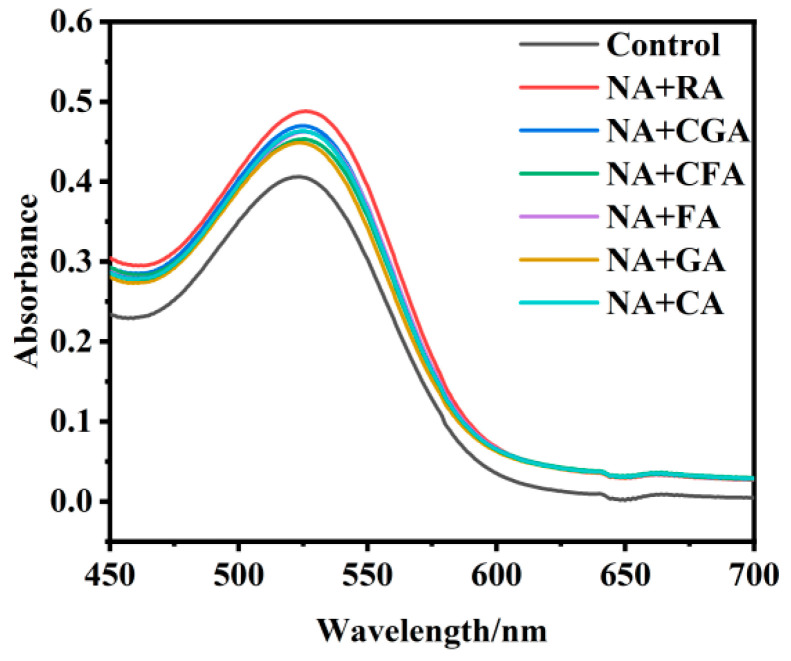
Visible spectra of RHA–copigment complexes after adding mixed copigments of NA and six other phenolic compounds.

To further enhance copigmentation efficiency, binary composite copigmentation systems were rationally constructed. NA, which exhibited the most prominent single-component copigmentation performance, was compounded with the six other phenolic acids at the fixed optimal molar ratio of 1:40. As depicted in Figure 3, all NA–phenolic acid binary composite groups achieved significantly improved spectral responses and better color protection effects compared with the individual copigment groups and the blank control, clearly verifying the existence of positive enhanced copigmentation interactions between different phenolic molecules. Among all the combined systems, the NA+RA binary complex exhibited the most outstanding reinforcement effect. Specifically, the maximum absorbance at λ_max_ increased by 20.98% relative to the pure RHAs control group, which was statistically superior to all other combined copigmentation systems (*p* < 0.05). Meanwhile, a bathochromic shift of 2–3 nm was observed, further confirming enhanced complex formation. The enhanced copigmentation performance of binary systems may be associated with potential cooperative intermolecular interactions. Specifically, NA, with its extended conjugated structure, could provide a strong π–π stacking framework, while phenolic acids such as RA might offer additional hydrogen bonding sites, which could help to stabilize the copigmentation complex. Such a complementary interaction pattern may contribute to the overall binding affinity and structural stability of the complexes. Similar enhancement phenomena between flavonoids and phenolic acids in anthocyanin stabilization have been documented in previous studies. For instance, Khalifa et al. [31] found that the combined application of quercetin and chlorogenic acid exerted a better color protection effect on anthocyanins than single phenolic additives. Consistent with the previous literature, our results further imply that the combined copigmentation efficiency of composite systems depends not only on the intrinsic molecular properties of individual copigments, but also on the structural complementarity and force matching degree between different phenolic components.

### 3.4. Thermodynamic Parameter Analysis of Copigmentation Reactions

The thermodynamic parameters of RHA–copigment copigmentation reactions, including the stoichiometric ratio (n), binding constant (K), and Gibbs free energy change (ΔG) for the copigmentation reactions between RHAs and different copigments at 20 °C are summarized in Table 3. The stoichiometric coefficient (n) reflects the binding molar proportion between copigments and anthocyanins during complex assembly [32]. Thermodynamic fitting results showed that NA possessed the maximum stoichiometric value (*n* = 0.9285), indicating that NA molecules could efficiently participate in ordered intermolecular assembly and form well-matched binding units with RHAs’ flavylium cations. In comparison, the *n* values of the remaining six phenolic copigments ranged only from 0.182 to 0.451, implying a relatively low binding compatibility and weak intermolecular coordination ability with RHAs. These differences may be attributed to the distinct molecular skeleton configurations, substituent group distributions, and spatial conformational differences among various phenolic copigments [14]. The binding constant (*K*) is an important thermodynamic index for evaluating the intermolecular affinity and complex structural stability. A higher *K* represents a stronger non-covalent binding force and more thermodynamically stable copigmentation complexes [33]. In accordance with the stoichiometric results, NA exhibited the highest binding constant (K = 9.24) in the thermodynamic fitting model, further verifying its optimal intermolecular affinity and outstanding complex stabilization capacity with RHAs. Importantly, this thermodynamic conclusion was highly consistent with the spectral experimental phenomena obtained in Section 3.2, where NA exhibited the strongest copigmentation effect, confirming that enhanced color intensity is closely associated with increased binding affinity.

Furthermore, the Gibbs free energy change (ΔG) was calculated to evaluate the thermodynamic spontaneity of the copigmentation reaction. Generally, a negative ΔG value indicates that the binding process proceeds spontaneously, while a more negative ΔG represents a stronger thermodynamic driving force and more favorable complexation behavior [14,34]. In the present study, NA, RA, CA, and FA yielded negative ΔG values, indicating that their intermolecular interactions with RHAs were thermodynamically spontaneous. In contrast, CGA, GA, and CFA presented positive ΔG values, implying non-spontaneous binding behavior and relatively weak interaction potential under the current experimental conditions. Notably, NA exhibited the lowest ΔG value (−5.4205 kJ/mol), indicating its optimal thermodynamic spontaneity and that it had the most favorable binding behavior with RHAs, which was highly consistent with the fact it had the highest *K* value and an optimal spectral copigmentation performance. The data consistency across thermodynamic and spectroscopic data further clarified that the copigmentation efficiency was mainly determined by the intensity of non-covalent intermolecular forces. In addition, copigments with extended π-conjugated skeletons and abundant active functional groups (e.g., hydroxyl groups) were more likely to form rigid and stable composite complexes. These findings were consistent with previous anthocyanin copigmentation-related thermodynamic research conclusions reported by Molaeafard et al. [34].

### 3.5. Effect of pH on Copigmentation Effects

**Figure 4:**
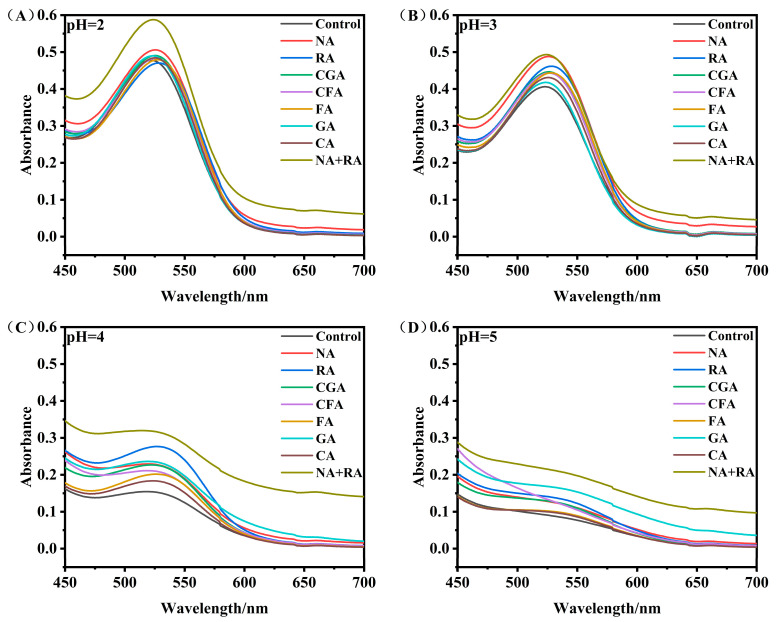
Visible spectra of various RHA–copigment complexes at different pH condition ((**A**): pH 2, (**B**): pH 3, (**C**): pH 4, (**D**): pH 5).

Figure 4 illustrates the visible absorption spectra of RHAs in the presence of different copigments over a pH range of 2.0–5.0. For the blank control sample, both the maximum absorption wavelength (λ_max_) and corresponding absorbance exhibited a trend of initial elevation followed by gradual attenuation as the ambient pH increased, with the optimal spectral response λ_max_ 523 nm occurring at pH 3.0. At pH 5.0, the characteristic absorbance intensity decreased sharply and the visible fingerprint absorption peak of anthocyanins almost completely faded, indicating significant color loss. After the addition of copigments, all experimental groups still exhibited a gradual reduction in spectral absorbance with increasing pH value, whereas the overall color retention ability significantly improved compared with the control. Specifically, at pH 2.0–3.0, the copigmentation effect was pronounced and the ΔA_λmax_ of each group ranged from 0.21% to 23.01%, accompanied by an obvious bathochromic shift of 1–6 nm. Under strongly acidic conditions, RHA molecules mainly exist in the stable flavylium cation configuration, which possesses a rigid planar π-conjugated skeleton. This highly ordered spatial structure provides favorable preconditions for strong π–π stacking assembly and hydrogen bonding with surrounding phenolic copigments, thereby effectively consolidating the composite complex structure and strengthening the copigmentation effect [35]. In contrast, when the pH value increased to 4.0–5.0, all samples presented prominent absorbance attenuation, obvious spectral peak broadening, and a slight hypsochromic shift of 2–3 nm. Such undesirable spectral deterioration was essentially attributed to the pH-triggered structural isomerization of anthocyanin molecules. Specifically, flavylium cations are gradually transformed into colorless hemiketal and chalcone derivatives under elevated pH conditions, which disrupts the complete B-ring conjugated system, weakens intermolecular force coordination, and ultimately impairs the binding affinity between RHAs and copigments [9,36]. Among all tested copigments, NA exhibited the strongest copigmentation effect at pH 2.0–3.0. This pH-adaptive stabilization performance was mainly attributed to the fact that both NA and RHAs belong to flavonoid derivatives with multiple hydroxyl groups, which can provide a sufficient number of binding sites for intermolecular interactions. The structural characteristics of NA, along with the pH-responsive structural transformation of anthocyanins under acidic conditions, contribute to the non-covalent interactions between NA and RHAs. These observations support that copigmentation is structure-dependent and highly sensitive to environmental pH, which regulates the structural configuration of anthocyanins and further affects their binding capacity with copigments.

### 3.6. Temporal Stability Analysis of RHA–Copigmentation Complexes During Dark Storage

**Figure 5:**
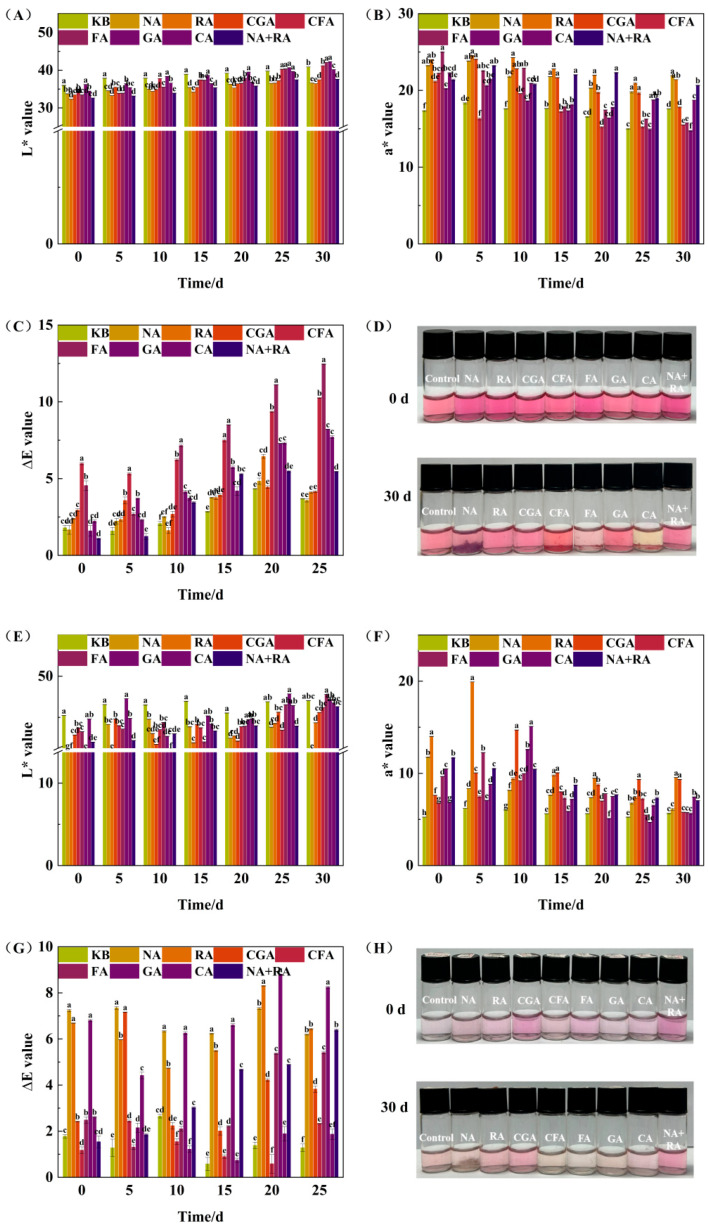
Color parameters and visible color changes of D3G and D3,5G with seven copigments after 30-day storage at 25 °C. Different lowercase letters indicate significant differences between samples (*p* < 0.05). (**A**–**D**) represent L*, a*, ΔE values, and visible color of D3G-based copigment complexes. (**E**–**H**) represent L*, a*, ΔE values, and visible color of D3,5G-based copigment complexes.

To further evaluate the stabilizing effects of the screened copigments on individual anthocyanin components, the two most abundant anthocyanin monomers (D3G and D3,5G) in red Huajiao were selected to construct simplified anthocyanin–copigment model systems. Changes in color parameters (L*, a*, and ΔE) were monitored during 30 d of storage at 25 °C in the dark. For the D3G-based, single-anthocyanin systems (Figure 5A–D), the L* and ΔE values gradually increased, while the a* values decreased over the storage period, indicating progressive color fading. This behavior is primarily attributed to the structural rearrangement of anthocyanin flavylium cations into colorless chalcone forms during storage [37]. Although exogenous phenolic copigments initially exerted effective color protection, their stabilizing efficacy gradually weakened as the storage time extended, suggesting the partial dissociation of copigment–anthocyanin complexes. Notably, the experimental groups supplemented with NA, CFA, FA, and CA exhibited visible precipitation phenomena after 30 d of storage, accompanied by obvious macroscopic color deterioration. Nevertheless, their color intensity remained significantly higher than that of the control group (*p* < 0.05), indicating that copigmentation also provided residual protection against continuous anthocyanin degradation. For the D3,5G-based model systems (Figure 5E–H), the overall variation trend of L* was consistent with that of D3G, showing a gradual increase during storage; however, the dynamic changes in a* and Δ*E* presented obvious non-monotonic fluctuation characteristics. In particular, the groups supplemented with RA, CA, and CGA exhibited a moderate increase in a* values within the first 15 days, followed by a remarkable decline in the subsequent storage stage. This suggests that the formation of copigment–anthocyanin complexes in the early stages was higher than the intrinsic degradation rate of anthocyanin. As the storage time was prolonged, the spontaneous chemical degradation of anthocyanins gradually dominated the reaction equilibrium, leading to irreversible color attenuation in the later stage [38]. Comparative analysis showed that the addition of RA, CGA, CA, and the optimal binary NA+RA composite copigment maintained vivid and stable color characteristics compared to the control systems (*p* < 0.05). Correspondingly, the final a* values of the above four groups reached 9.55, 9.34, 7.43, and 7.04, respectively, further verifying the reliable short-term color-preserving capacity of optimized copigmentation systems for D3,5G monomers within a storage period of less than 15 days. Importantly, distinct temporal stability differences between D3G and D3,5G model systems indicated that the anthocyanin structure significantly influences the copigmentation stability. The additional glycosyl substitution on the C5 position of the D3,5G molecular skeleton effectively changed the spatial steric hindrance distribution and intermolecular force matching mode, thus resulting in completely different dynamic stability behaviors during long-term storage. Overall, the copigmentation stabilization strategy can effectively delay anthocyanin fading and improve color stability, especially in the early storage stages.

### 3.7. FT-IR Analysis

**Figure 6:**
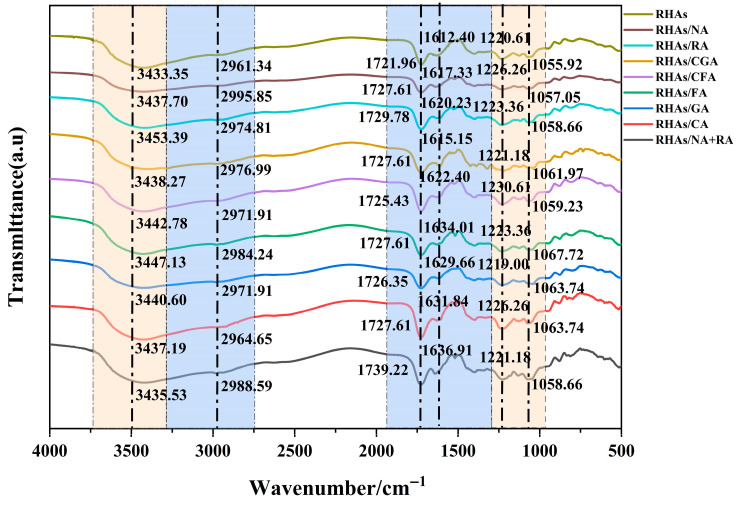
FTIR spectra of RHAs and RHA–copigment complexes.

FT-IR spectroscopic detection was performed to characterize the potential intermolecular interactions between RHAs and selected copigments, and the collected spectra are presented in Figure 6. The broad and strong absorption band near 3430 cm^−1^ was attributed to the stretching vibrations of hydroxyl groups (-OH), which originated from phenolic hydroxyl groups, glycosidic hydroxyl structures, and hydrogen bonds. The characteristic absorption peaks at approximately 2980 cm^−1^ and 1720 cm^−1^ corresponded to C–H and C=O stretching vibrations, respectively, while the distinct spectral band centered at 1620 cm^−1^ was assigned to the stretching vibrations of aromatic conjugated C=C bonds. The weak peak detected at 1220 cm^−1^ was derived from in-plane C-H bending vibrations, C=O stretching vibrations, C-C skeletal vibrations, and pyran ring stretching vibrations, all of which are typical fingerprint spectral characteristics of flavonoid compounds [14]. In addition, after copigment incorporation, the absorption peak near 1050 cm^−1^ was attributed to the bending vibrations of the C-O-C groups. Notably, the bands around 3433 cm^−1^ assigned to O–H stretching shifted to higher wavenumbers, ranging from 3447 cm^−1^ to 3435 cm^−1^. This shift may indicate changes in hydrogen bond environments, suggesting the formation of intermolecular hydrogen bonds between anthocyanins and copigments. Collectively, all subtle but regular spectral variations in functional group regions suggested the occurrence of non-covalent intermolecular association behaviors, further implying that hydrogen bonding may act as one of the major driving forces in stabilizing the copigmentation composite structures.

### 3.8. Molecular Docking

**Figure 7:**
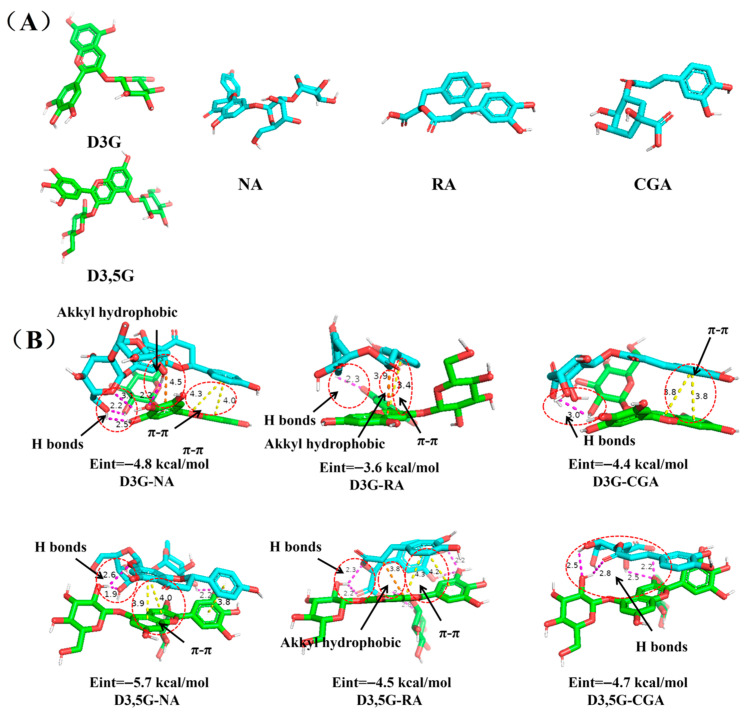
Three-dimensional molecular models of D3G, D3,5G, and different copigments (NA, RA, and CGA) (**A**), molecular docking and binding conformations of D3G and D3,5G with copigments in the 3D docking models (**B**).

In order to elucidate the detailed molecular interactions of RHAs with various copigments, molecular docking was performed. The two dominant anthocyanin monomers (D3,5G and D3G) and three high-efficiency copigments (NA, RA, and CGA) screened in Section 3.2 were selected to construct molecular docking models. The optimal binding conformations and corresponding interaction energy parameters of all docking complexes are displayed in Figure 7. The docking results suggest that the intermolecular interactions between anthocyanins and copigments were mainly via hydrogen bonds, π–π stacking, and alkyl hydrophobic interactions, which was consistent with the previous FT-IR experimental findings. According to the calculated binding energy data, the interaction strength sequence of different copigments with the D3G monomer was as follows: D3G-NA (−4.8 kcal/mol) > D3G-CGA (−4.4 kcal/mol) > D3G-RA (−3.6 kcal/mol). Meanwhile, the binding affinity ranking for the D3,5G monomer was determined as D3,5G-NA (−5.7 kcal/mol) > D3,5G-CGA (−4.7 kcal/mol) > D3,5G-RA (−4.5 kcal/mol). Obviously, NA presented the lowest binding energy and the strongest molecular affinity in both anthocyanin monomer systems, supporting its better copigmentation stabilization ability. The superior binding performance of NA is closely related to its extended conjugated structure and abundant surface hydroxyl functional groups, which effectively enlarged the molecular contact area and provided a sufficient number of binding sites for intermolecular hydrogen bond formation [39]. Collectively, the molecular docking results provide supportive evidence for the experimental findings, suggesting that the strength of copigmentation may be associated with the combined effects of molecular planarity, conjugation, and functional group availability. Combined with spectroscopic characterization, thermodynamic fitting, and molecular simulation evidence, the present study systematically suggests that the anthocyanin copigmentation process is a structure-dependent and dynamically regulated behavior that is comprehensively modulated by the intrinsic molecular configuration of anthocyanins, the structural characteristics of phenolic copigments, and the external environmental conditions.

### 3.9. Effects of Different CGA Concentrations on the Storage Quality of Red Huajiao

Based on the strong copigmentation effect and stability of the RHA–CGA complex, CGA was selected as a promising protective additive for the color preservation of red Huajiao. The practical preservation potential of gradient CGA pretreatment in maintaining the overall commercial quality of fresh red Huajiao during frozen storage is further systematically evaluated in this section.

#### 3.9.1. Sensory Quality

**Figure 8:**
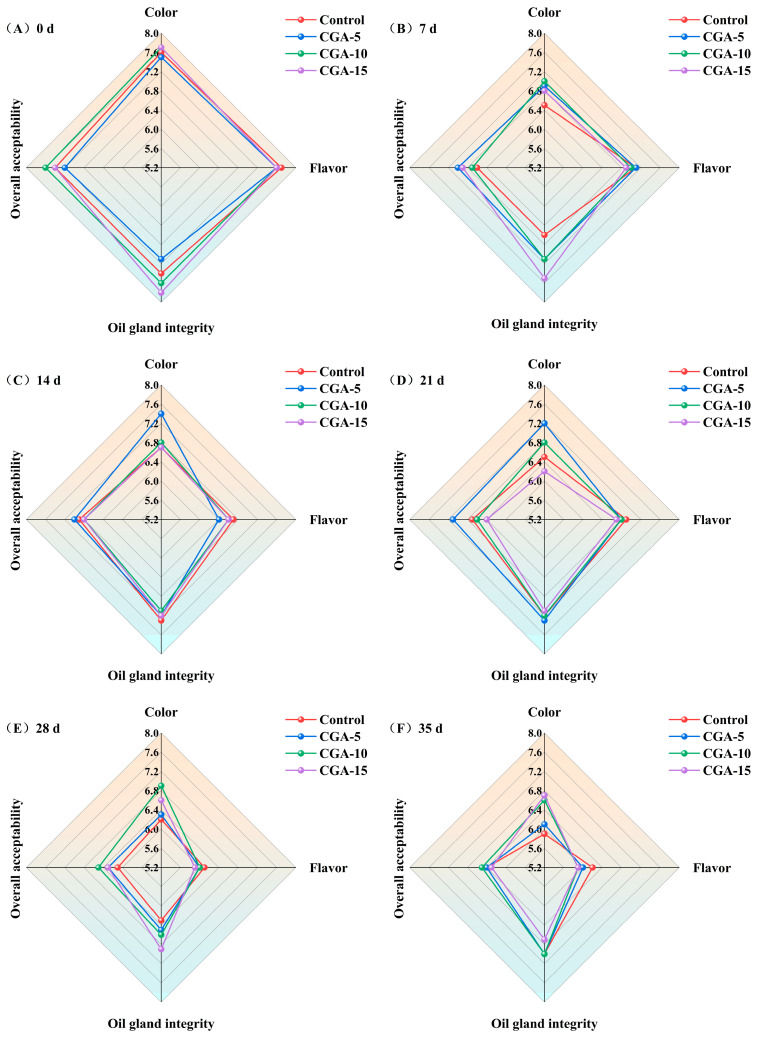
Sensory radar diagram of red Huajiao pretreated with different concentrations of CGA during storage at −18 °C. (**A**–**F**) represent storage days 0, 7, 14, 21, 28 and 35, respectively.

Figure 8 illustrates the sensory changes in red Huajiao during frozen storage. Overall, the sensory attributes, including the surface color, characteristic flavor, and oil gland integrity gradually deteriorated with increasing storage time. Compared with the control group, CGA-treated samples maintained a better color retention performance throughout the storage period. No obvious differences in the overall flavor profiles were detected among any of the groups within the first 28 days of frozen storage (Figure 8A–E). At the end of storage (day 35), the high-concentration CGA groups exhibited a slight decrease in flavor scores, whereas the control group retained relatively preferable flavor characteristics (Figure 8F). According to Figure 8A,B, the 15 mmol/L CGA group maintained optimal oil gland integrity during the early storage stage (0–14 d), followed by the 10 mmol/L CGA group. From 14 to 35 d (Figure 8C–F), no regular or significant differences in oil gland integrity were observed between the CGA-treated groups and the control. Nevertheless, all CGA pretreatment samples generally presented higher residual oil gland integrity than the control group throughout the whole storage process. In particular, the 10 mmol/L CGA group obtained the highest comprehensive sensory acceptability score among all the experimental groups. Notably, the sensory evaluation in this study involved a small sensory panel consisting of individuals within a limited age range of 20 to 25 years. These limitations in both the size of the panel and its age distribution may restrict the generalizability of the sensory results considering that sensory perceptions of color, flavor, and texture can vary greatly across different age groups and larger population samples. In future, a larger and more diverse sensory panel with a broader age range will be employed to enhance the reliability and practical applicability of sensory evaluation conclusions.

#### 3.9.2. Color Stability and Multivariate Statistical Discrimination Analysis

**Figure 9:**
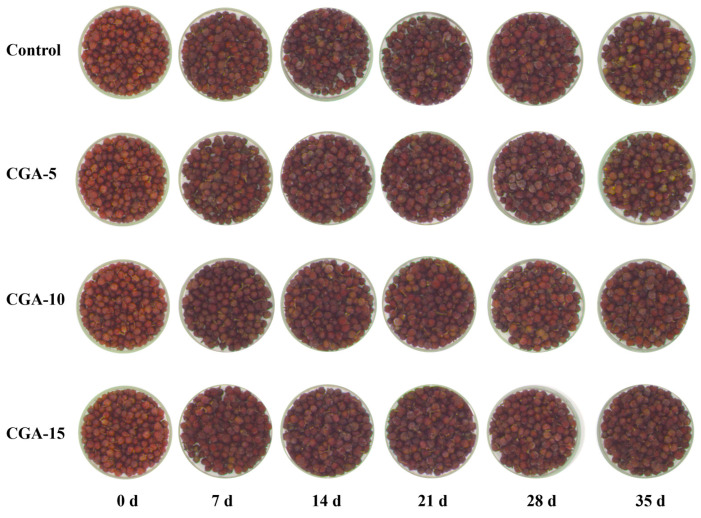
Appearance of red Huajiao pretreated with different concentrations of CGA during storage at −18 °C.

The appearance and continuous color dynamic changes in red Huajiao with gradient CGA pretreatment were monitored during a 35-day frozen storage period, and the representative visual comparison images are displayed in Figure 9. Fresh red Huajiao on day 0 presented uniform luster, full grain morphology, and an intact pericarp structure, with highly stable surface color characteristics. With the extension of the frozen storage time, the overall quality of appearance and surface color attributes of red Huajiao in each group gradually deteriorated. After 28 days of storage, the control group exhibited severe symptoms of quality deterioration, including obvious surface browning, local whitening spots, and irreversible pericarp cracking. In contrast, all CGA-treated groups maintained preferable surface color retention, a complete grain structural morphology, and well-preserved oil gland integrity. These findings suggest that appropriate CGA pretreatment effectively improves the frozen storage stability of fresh red Huajiao, significantly slowing down quality deterioration over the storage period.

**Figure 10:**
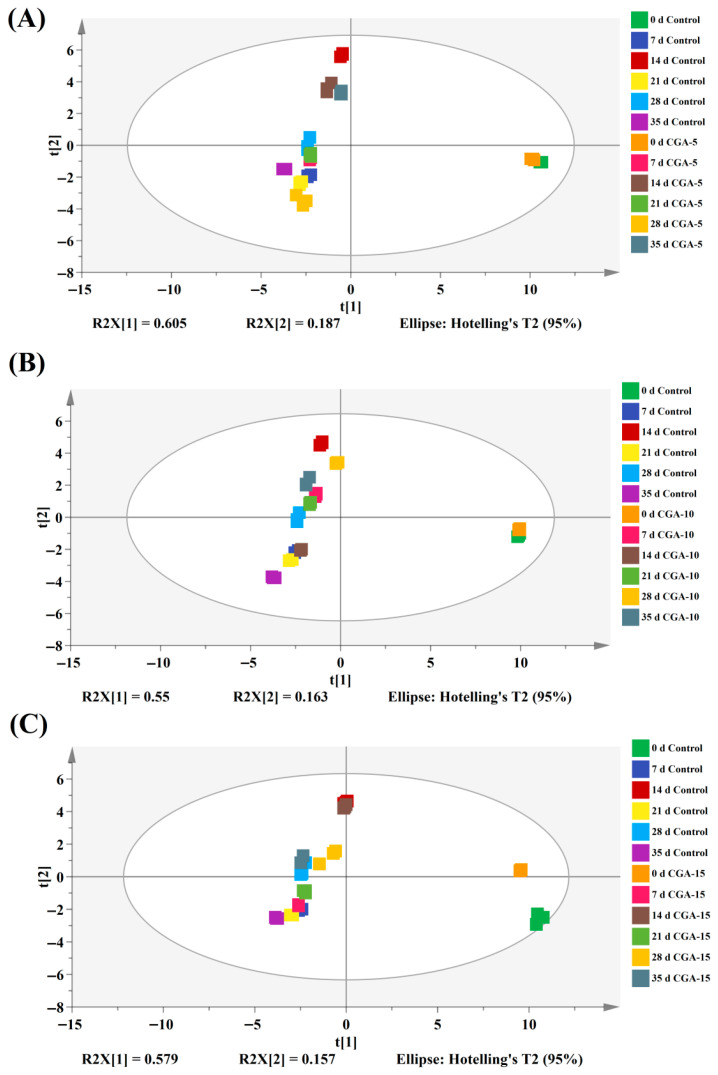
PLS-DA score graphs of red Huajiao pretreated with different concentrations of CGA during storage at −18 °C. (**A**) 5 mmol/L CGA treatment, (**B**) 10 mmol/L CGA treatment, and (**C**) 15 mmol/L CGA treatment.

To further quantitatively distinguish the subtle color differences induced by CGA dosage and storage time, all color parameter datasets collected at different storage stages were integrated to construct standardized multivariate data matrices, and partial least squares discriminant analysis (PLS-DA) was subsequently performed. The corresponding statistical classification results are shown in Figure 10. As a classical supervised multivariate statistical method, PLS-DA can maximize the inter-group spatial divergence under artificial classification conditions, achieving more accurate sample discrimination than unsupervised PCA analysis, and thus it is highly suitable for the complex differential screening of food color data. In the PLS-DA two-dimensional score plot, samples with closer spatial coordinates indicate higher similarity in overall color characteristics. As illustrated in Figure 10A, the control red Huajiao stored for 35 days was distant from the initial day 0 fresh baseline group, indicating remarkable color differentiation and severe color degradation. In comparison, the 5 mmol/L CGA-treated samples still maintained high similarity to the day 0 fresh state after 21 and 28 days of frozen storage, showing effective color maintenance effects. Likewise, Figure 10B further verifies that the control group exhibited the most significant color deviation from the fresh baseline at the later storage stage (day 35). Among all treatment groups, the 10 mmol/L CGA group retained the most stable original color characteristics and the closest spatial similarity to the fresh samples at day 28. Contrastingly, the color protection of the 15 mmol/L CGA group was only effective within the initial 7 days of early storage, and its stabilization efficiency gradually weakened thereafter. These results suggest that CGA pretreatment effectively hindered the irreversible color deterioration of red Huajiao during frozen storage, and 10 mmol/L CGA was identified as the optimal concentration for the color maintenance of frozen red Huajiao.

**Figure 11:**
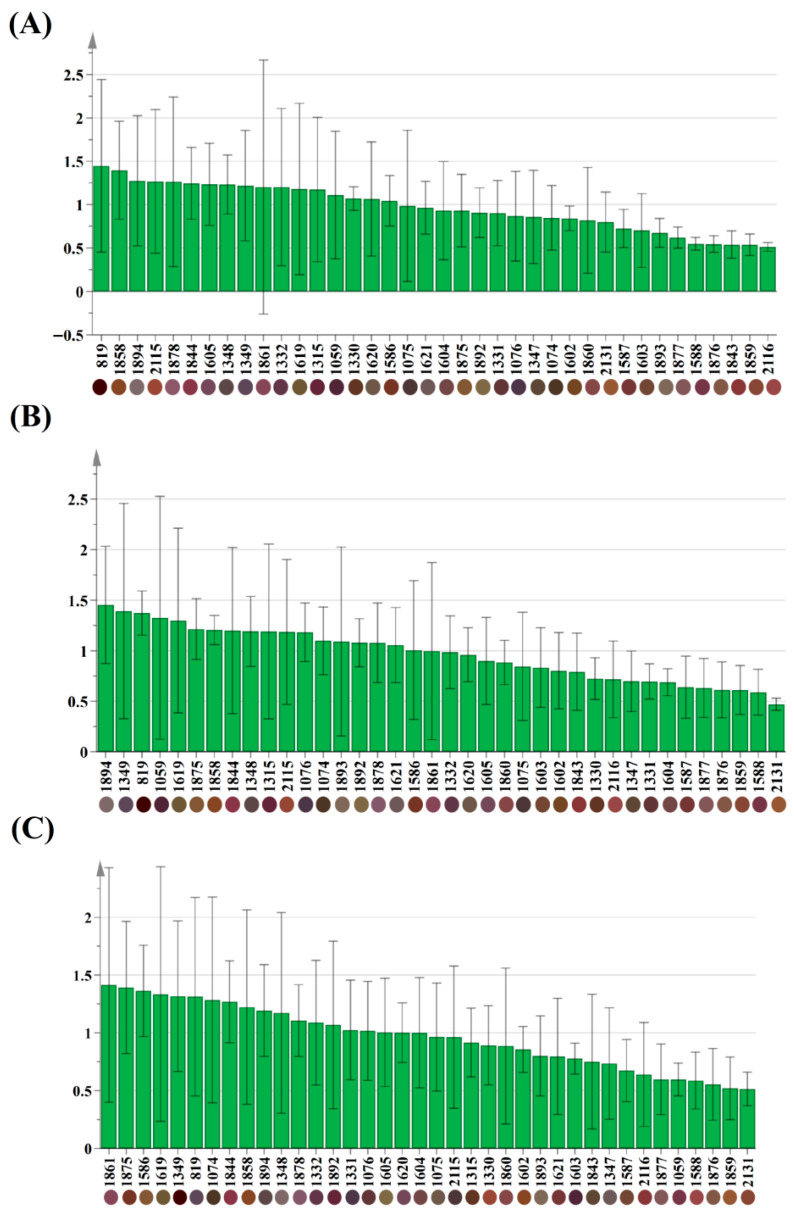
The contributions of the main color value variables of red Huajiao pretreated with different concentrations of CGA during storage at −18 °C. (**A**) 5 mmol/L CGA-treated samples, (**B**) 10 mmol/L CGA-treated samples, and (**C**) 15 mmol/L CGA-treated samples.

Variable importance in projection (VIP) derived from the PLS-DA model was further calculated to screen the key differential color indicators dominating the surface color of red Huajiao. In general, color variables with VIP values greater than 1.0 are regarded as core characteristic indicators, with significant discriminatory contributions to sample classification, and higher VIP values represent stronger regulatory effects on overall color fluctuation [19]. As illustrated in Figure 11, five critical color feature parameters with VIP values exceeding 1.0 were successfully screened out, sequentially defined as 1075 (R 72, G 56, B 56), 1331 (R 88, G 56, B 56), 1621 (R 104, G 88, B 88), 1861 (R 120, G 72, B 88), and 1893 (R 120, G 104, B 88). These findings suggest that these five color indicators significantly influence the color variations in CGA-treated preserved red Huajiao across different storage periods. The detailed RGB values of the remaining non-significant color parameters are listed in Supplementary Table S2.

#### 3.9.3. Flavor Characteristics

**Figure 12:**
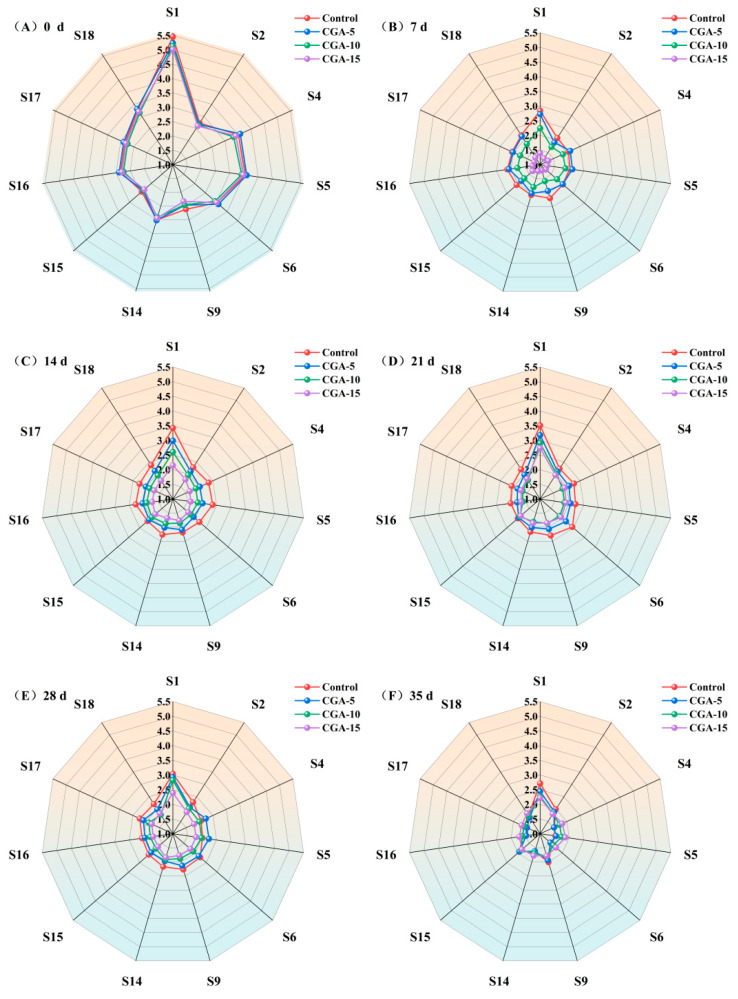
E-nose signal radar diagram of CGA-pretreated red Huajiao during storage at −18 °C. (**A**–**F**) represent storage days 0, 7, 14, 21, 28 and 35, respectively.

E-nose analysis was applied to rapidly characterize the dynamic variations in the overall volatile flavor fingerprints of red Huajiao under different CGA treatments and frozen storage durations, and the response intensity distributions of multiple sensors are displayed in Figure 12. As shown in Figure 12A, red Huajiao showed obvious responses to sensors S1 and S9, which are sensitive to hydrocarbon substances and aldehyde and ketone alcohol volatile fractions, respectively. These sensor-sensitive volatile fractions are closely related to the characteristic flavor properties of red Huajiao [10]. Notably, compared with the initial fresh samples on day 0, the flavor profile of red Huajiao deteriorated markedly from day 7 to day 35, suggesting that the frozen environment caused adverse effects on the intrinsic volatile flavor substances of red Huajiao. Furthermore, across the later storage period of 7–35 d (Figure 12B–F) red Huajiao exhibited the most prominent response to sensor S1. Although a decreasing trend in flavor intensity was observed with increasing concentrations of CGA, no statistically significant differences were noted (*p* < 0.05). The above results indicate that exogenous CGA pretreatment exerted no substantial adverse interference or destructive effects on the original flavor attributes of red Huajiao. Accordingly, the irreversible flavor attenuation during long-term frozen storage was reasonably attributed to the physical freezing damage to the surface oil gland tissues of red Huajiao pericarp. Nevertheless, it is important to acknowledge that the E-nose analysis only provided an overall fingerprint of volatile odor profiles without precise qualitative and quantitative identification of individual volatile compounds. To achieve a thorough characterization of volatile substances, upcoming research will integrate gas chromatography-based analytical methods for more focused analysis.

#### 3.9.4. Pungent Compounds

**Figure 13:**
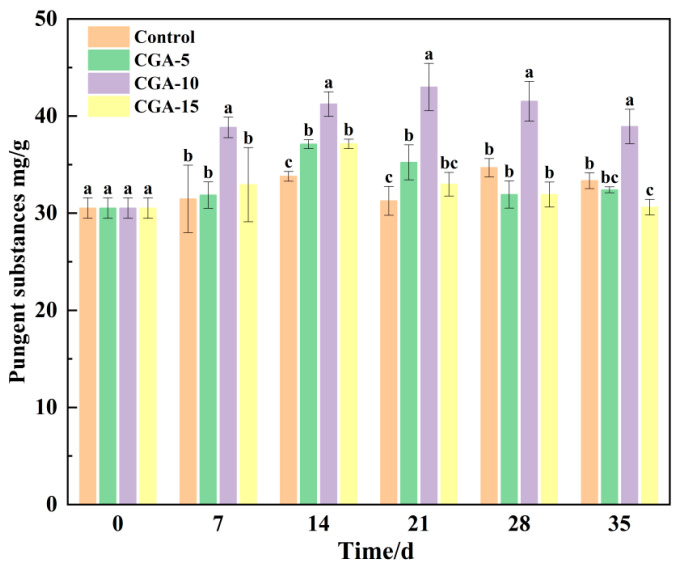
Pungent substance content of CGA-pretreated red Huajiao during storage at −18 °C. Lowercase letters represent statistically significant differences at *p* < 0.05.

Figure 13 illustrates the changes in the total content of pungent substances in red Huajiao pretreated with various concentrations of CGA during storage. As shown in Figure 13, the content of total pungent substances exhibited a regular dynamic pattern of initial elevation followed by gradual reduction, with the maximum level observed on day 21 of frozen storage. Notably, among all experimental groups, the 10 mmol/L CGA group consistently maintained the highest content of total pungent compounds throughout the storage period. The initial increase in the pungent substances content may be attributed to variations in the moisture content of red Huajiao during storage. These findings suggest that CGA can preserve both sensory-active compounds and the surface visual quality while maintaining the intrinsic characteristic pungency of red Huajiao. The UV spectrophotometric method used in this study allows for the rapid estimation of the total pungent substances, but provides lower specificity compared to chromatographic techniques for the targeted quantification of individual sanshool monomers. The specific chemical composition of the pungent compounds requires further analysis using the liquid chromatography method.

## 4. Conclusions

In this work, the copigmentation behaviors and underlying interaction mechanisms between red Huajiao anthocyanins (RHAs) and seven representative phenolic compounds were systematically investigated through multi-dimensional technical systems, including spectroscopic characterization, thermodynamic fitting, molecular docking simulation, and practical frozen storage application validation. The results revealed that RHAs were predominantly delphinidin derivatives, accounting for 80.84% of the total anthocyanins. Exogenous phenolic copigment supplementation significantly enhanced the color intensity and stability of RHAs, and the copigmentation performance was strongly determined by the inherent molecular structural characteristics of individual phenolic molecules. Among all copigments, NA exhibited the most prominent copigmentation capacity, mainly due to its extended conjugated skeleton and abundant surface hydroxyl substituents, which effectively facilitated intermolecular π–π and hydrogen bond cross-linking with flavylium cations. Thermodynamic analysis further suggested that the intermolecular binding interactions between RHAs and four optimal copigments (NA, RA, FA, and CA) belonged to spontaneous exothermic reactions, which were mainly caused by structural matching and non-covalent force coordination. Environmental factors further modulated the copigmentation behavior of RHAs. In particular, pH influenced the structural state of anthocyanins, thereby affecting their interaction capacity with copigments, whereas the storage duration dynamically regulated the equilibrium between complex formation and degradation. Combined FT-IR spectroscopy and molecular docking consistently suggested that non-covalent forces including hydrogen bonds, π–π stacking, and hydrophobic interactions dominated the copigmentation process, while molecular planarity and substituent groups collectively determined the binding strength. Practical preservation tests suggested that 10 mmol/L chlorogenic acid (CGA) treatment effectively maintained the postharvest quality of frozen red Huajiao, retaining a stable color appearance, an intact oil gland structure, and the characteristic pungent components. Overall, phenolic copigmentation is a structure-sensitive and environment-responsive strategy for anthocyanin protection. This study provides fundamental mechanistic insights for developing efficient natural color stabilizers for maintaining the storage quality of frozen anthocyanin-rich spices.

## Figures and Tables

**Table 1:** Composition and relative content of anthocyanin monomers.

No.	RT/min	Anthocyanin Monomers	Molecular Formula	[M]+ (*m*/*z*)	MS/MS (*m*/*z*)	Relative Content (%)
1	13.74	Cyanidin-3-O-lucoside	C_21_H_21_O_11_	449.10	287.05	2.62
2	14.65	Cyanidin-3-rutinoside	C_27_H_31_O_16_	595.17	287.05	3.43
3	17.69	Delphinidin-3-O-rutinoside-7-O-glucoside	C_33_H_41_O_21_	773.21	611.16/465.10/303.05	12.63
4	18.66	Delphinidin-3,5-diglucoside	C_27_H_31_O_17_	627.15	465.11/303.05	28.23
5	22.63	Delphinidin-3-O-rutinoside	C_27_H_31_O_16_	611.16	465.11/303.05	11.04
6	23.01	Delphindin-3-O-galactoside	C_21_H_21_O_12_	465.10	303.05	13.24
7	23.40	Delphindin-3-O-glucoside	C_21_H_21_O_12_	465.10	303.05	14.86
8	26.33	Petunidin-3-O-rutinoside	C_28_H_33_O_16_	625.18	479.12/317.07	8.63
9	26.55	Delphinidin-3-O-rhamnoside	C_21_H_21_O_11_	449.11	303.05	3.00
10	27.38	Petunidin-3-O-glucoside	C_22_H_23_O_12_	479.11	317.07	2.32

**Table 2:** Maximum absorption wavelength (λ_max_), color-enhancing effect (ΔA), and bathochromic shift (Δλ_max_) of the copigmented complexes of RHAs with different copigments.

Copigments	Molar Ratio of RHAs to Copigments	λ_max_ (nm)	ΔA (%)	Δλ_max_ (nm)
NA	1:1	523	2.96	0
	1:5	523	5.08	0
	1:10	523	4.74	0
	1:20	523	13.28	0
	1:40	524	19.46	1
RA	1:1	524	2.54	1
	1:5	525	4.14	2
	1:10	526	5.75	3
	1:20	526	8.71	3
	1:40	528	13.87	5
CGA	1:1	523	2.03	0
	1:5	523	2.79	0
	1:10	523	3.21	0
	1:20	525	5.92	2
	1:40	526	12.01	3
CFA	1:1	523	3.47	0
	1:5	523	4.57	0
	1:10	523	4.60	0
	1:20	524	5.57	1
	1:40	526	7.10	3
FA	1:1	523	1.35	0
	1:5	524	1.94	1
	1:10	524	3.38	1
	1:20	526	5.75	3
	1:40	528	10.4	5
GA	1:1	523	1.01	0
	1:5	523	1.52	0
	1:10	523	2.20	0
	1:20	523	2.62	0
	1:40	523	4.91	0
CA	1:1	523	2.11	0
	1:5	523	2.20	0
	1:10	525	4.14	2
	1:20	526	4.31	3
	1:40	526	7.36	3

**Table 3:** Thermodynamic properties of RHAs combined with different phenolic copigments.

Copigments	F(x)	R^2^	n	K	∆G (KJ/mol)
NA	y = 0.3583x + 0.9276	0.9648	0.9285	9.2433	−5.4205
RA	y = 0.2354x + 0.0087	0.9960	0.2345	1.0087	−0.0212
CGA	y = 0.2710x − 0.1069	0.9442	0.2710	0.8986	0.2606
CFA	y = 0.2941x − 0.1998	0.9221	0.2941	0.8189	0.4870
FA	y = 0.4510x + 0.3583	0.9919	0.4510	1.4310	−0.8733
GA	y = 0.1820x − 0.8842	0.9538	0.1820	0.4130	2.1551
CA	y = 0.9285x + 2.2239	0.9210	0.3583	2.5284	−2.2609

## Data Availability

The original contributions presented in this study are included in the article/Supplementary Materials. Further inquiries can be directed to the corresponding author.

## Supplementary Materials

The following supporting information can be downloaded at: https://www.mdpi.com/article/10.3390/foods15101719/s1. Table S1. Sensory quality scoring criteria for red Huajiao. Table S2. Values of *L*, *a*, *b* and R, G, B for different color indicators. Figure S1. MS/MS secondary fragmentation spectra and proposed structures of anthocyanin monomers of RHA monomers.
