# Supplementary material for: Phenolic Copigment Enhanced Anthocyanin Stability and Color Intensity of Frozen Red Huajiao (Zanthoxylum bungeanum Maxim.) Fruit
Source: Foods. 2026 May 13;15(10):1719. doi: 10.3390/foods15101719 (PMC13205320; doi:10.3390/foods15101719)
Supplement: Supplementary file 1 [file foods-15-01719-s001.zip › foods-4277852-supplementary.pdf]

Supplementary Information

Phenolic copigments enhanced anthocyanin stability and color intensity of frozen-storage red Huajiao (*Zanthoxylum bungeanum* Maxim.) fruit

Xin Yang [1], Yishan Chen [1], Xiao He [1], Jiani Liu [1], Shuang Xian [1], Shanshan Li [1], Xiaoyan Hou [1], Man Zhou [1], Qiang Cui [1], Jie Yu [1], Xiang Zhu [1], Zhiqing Zhang [1], Anjun Chen [1], Guanghui Shen [1,*]

1 College of Food Science, Sichuan Agricultural University, Ya'an, Sichuan, 625014, China ; 13550232902@163.com (X. Yang); 15334585653@163.com (Y.S Chen); 18175033825@163.com (X. He); 15242523815@163.com (J.N. Liu); xianshuang@stu.sicau.edu.cn (S. Xian); lss@sicau.edu.cn (S.S. Li); houxiaoyan106@163.com (X.Y Hou); zhouman@sicau.edu.cn (M. Zhou); cuiqiangwx@163.com (Q. Cui); jieyu0609@163.com (J. Yu); zxpaocai@163.com (X. Zhu); zqzhang721@163.com (Z.Q Zhang); chen_anjun@sicau.edu.cn (A.J. Chen).

* Correspondence: shenghuishen@163.com (G.H. Shen); Tel.: +86-0835-2882187

Supplementary Information

TableS1 Sensory quality scoring criteria for red Huajiao

Scoring items	Sensory quality description	Score range
Color	Bright red and lustrous appearance	7~10
	Dark red and dull luster	4~6
	Grey and lacks luster	1~3
Flavor	Normal pleasant aroma and no off-flavors	7~10
	The aroma is rather faint and a slight off-flavor	4~6
	No fragrance and a distinct unpleasant smell	1~3
Integrity of oil glands	The surface oil glands are intact and undamaged	7~10
	The oil glands on the surface are relatively intact and slightly damaged	4~6
	The surface oil glands are incomplete and severely damaged	1~3

Table S2 Values of *L*, *a*, *b* and R, G, B for different color indicator

No.	Color indicator	*L*	*a*	*b*	R	G	B
1	819	23.521	0	0	56	0	0
2	1059	20.838	17.262	-3.617	72	40	56
3	1074	24.969	5.435	12.857	72	56	40
4	1075	25.311	7.321	2.842	72	56	56
5	1076	25.772	9.768	-7.065	72	56	72
6	1315	23.446	24.046	0.554	88	40	56
7	1330	27.116	13.189	16.120	88	56	40
8	1331	27.425	14.678	6.172	88	56	56
9	1332	27.843	16.637	-3.73	88	56	72
10	1347	31.936	5.063	12.310	88	72	56
11	1348	32.280	7.008	2.680	88	72	72
12	1349	32.719	9.048	-6.851	88	72	88
13	1586	29.523	20.606	19.732	104	56	40
14	1587	29.800	21.795	9.880	104	56	56
15	1588	30.177	23.375	0.002	104	56	72
16	1602	33.678	11.290	24.684	104	72	40
17	1603	33.911	12.494	15.346	104	72	56
18	1604	34.229	14.099	5.757	104	72	72
19	1605	34.635	16.097	-3.770	104	72	88
20	1619	38.403	3.206	21.091	104	88	56
21	1620	38.671	4.784	11.872	104	88	72
22	1621	39.015	6.759	2.558	104	88	88
23	1843	32.378	28.555	13.862	120	56	56
24	1844	32.716	29.844	4.031	120	56	72
25	1858	35.894	18.738	27.913	120	72	40
26	1859	36.108	19.730	18.692	120	72	56
27	1860	36.400	21.060	9.162	120	72	72
28	1861	36.774	22.730	-0.35	120	72	88
29	1875	40.270	10.605	23.892	120	88	56
30	1876	40.521	11.939	14.735	120	88	72
31	1877	40.843	13.619	5.449	120	88	88
32	1878	41.238	15.634	-3.774	120	88	104

33	1892	44.936	2.918	20.515	120	104	72
34	1893	45.213	4.566	11.511	120	104	88
35	1894	45.555	6.553	2.461	120	104	104
36	2115	38.487	26.666	22.278	136	72	56
37	2116	38.754	27. 779	12.824	136	72	72
38	2131	42.329	17.853	26.952	136	88	56
39	2132	42.562	18.983	17.872	136	88	72
40	2133	42.862	20.415	8.624	136	88	88
41	2148	46.704	10.058	23.198	136	104	72
42	2149	46.353	8.102	39.790	136	104	40
43	2150	47.289	13.213	5.207	136	104	104
44	2166	51.589	4.390	11.206	136	120	104
45	2388	44.766	25.820	21.233	152	88	72
46	2389	45.045	27.046	12.035	152	88	88
47	2405	48.886	18.346	17.206	152	104	88

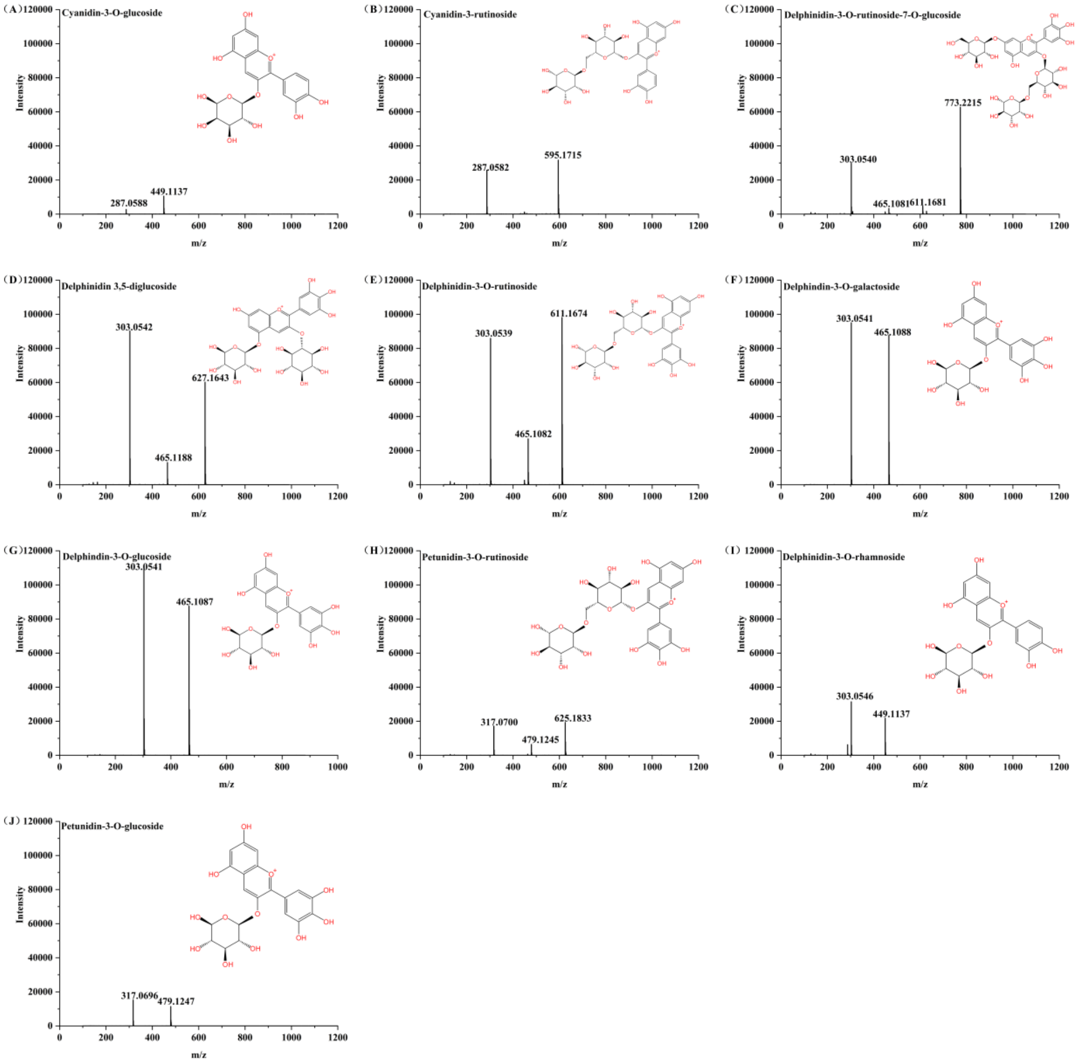

Fig. S1 MS/MS secondary fragmentation spectra and and proposed structures of anthocyanin monomers of RHA monomers
